# Editorial: Microorganisms in tea and tea beverages

**DOI:** 10.3389/fmicb.2024.1536704

**Published:** 2024-12-20

**Authors:** Chun Zou, Yu Xiao, Jasmina Vitas

**Affiliations:** ^1^Tea Research Institute Chinese Academy of Agricultural Sciences, National Engineering Research Center for Tea Processing, Hangzhou, China; ^2^College of Food Science and Technology, Hunan Agricultural University, Changsha, China; ^3^University of Novi Sad, Faculty of Technology Novi Sad, Novi Sad, Serbia

**Keywords:** tea and tea beverages, key microorganisms, screening and identification, flavor quality, health benefits, innovative processing technologies, intestinal microorganisms

Tea is one of the most popular and widely consumed beverages in the world, and microorganisms are closely related to the tea industry. On the one hand, microbial fermentation is considered a key factor in the formation of sensory quality and health benefits of fermented tea and tea beverages, such as dark tea, kombucha, tea wine, etc. On the other hand, microorganisms are closely related to the growth and development of tea plants and the formation of tea quality. Therefore, more and more research has been carried out on microorganisms in tea and tea beverages in recent years.

During the processing of fermented tea and tea beverages, the metabolism of microorganisms plays an important role in the formation of their flavor and health benefits. Due to the wide variety of microorganisms, it is necessary to detect and identify the key microorganisms in tea and tea beverages. In order to explore the impact of key microorganisms on the flavor quality and health benefits of fermented tea and tea beverages, metagenomics, metabolomics, and other methods can be comprehensively applied. On the basis of clarifying the role of microorganisms, new processing technologies can be developed to improve the quality of tea and tea beverages. In addition, the effects of soil microorganisms and endophytic microorganisms on the growth of tea plants and the quality of tea leaves are also worthy of further research.

In this context, the Research Topic on “*Microorganisms in Tea and Tea Beverages*” has been organized. This Research Topic collected six research articles from international researchers. This Research Topic aims to understand the role of microorganisms in tea and tea beverages, and promote the integration of microbiology and tea science.

Kombucha is a tea beverage fermented by a symbiotic culture of bacteria and yeast. Liao et al. systematically investigated microbial community succession and metabolite changes in a back slopping kombucha fermentation process using high-throughput long-read amplicon sequencing and ultraperformance liquid chromatography–mass spectrometry, respectively. Their study sheds light on the microbial and metabolite dynamics of kombucha fermentation, emphasizing the importance of microbial control and quality assurance measures in the production process.

The health benefits of kombucha tea have received widespread attention from researchers, especially its effect on gut microbiota. Su et al. offers deeper insights into the mechanisms underlying turmeric kombucha (TK) against sepsis by connecting bioactive metabolites, gut microbiota modulation, and anti-inflammatory effects. Their findings shed light on the protective effects and underlying mechanisms of TK in mitigating LPS-induced sepsis, highlighting TK as a promising anti-inflammatory agent and revealing new functions of kombucha prepared through traditional fermentation methods.

Pu-erh tea is a renowned post-fermented tea that is crafted in Yunnan Province, located in Southwest China. Li et al. examined the carbohydrate metabolism and expression of carbohydrate active enzyme genes during the fermentation of tea leaves with *Aspergillus luchuensis*. They discovered high expression of 40 genes encoding 16 carbohydrate enzymes during the fermentation process. Their findings provide insight into the metabolism of tea carbohydrates fermented with a dominant fungus and lay the groundwork for further understanding carbohydrate changes in Pu-erh tea fermentation.

The quality of Sichuan south-road dark tea (SSDT) is mainly determined by the microbial action during pile fermentation. Liu et al. comprehensively analyzed the air samples from SSDT pile fermentation plant by high-throughput sequencing. They revealed the presence of 2 and 24 phyla, 9 and 49 classes, 18 and 88 orders, 28 and 153 families, 38 and 253 genera, and 47 and 90 species of fungi and bacteria, respectively, across all samples. Their study paves the way for microbial traceability in piled SSDT during production and significantly advance the understanding of microbial influences on SSDT quality.

Miang is one of the post-fermented teas made in Northern Thailand. Horie et al. isolated a total of 1,181 *Lactobacillaceae bacteria* strains from Miang, of which 31 isolates exhibited antibacterial activity. Their research revealed that Miang contains lactic acid bacteria that could potentially be used as probiotics.

Microorganisms exhibit intricate interconnections with tea plants. Yao et al. collected rhizosphere soils and tender leaves of different varieties of tea plants from the same tea plantation and offered comprehensive analyses of microbial diversity across different tea varieties utilizing 16S rRNA gene and ITS amplicon sequencing techniques. Their study has shed light on the intricate relationships of tea plant varieties with their associated microbial communities, unveiling the importance of microorganisms and tea varieties with higher tea polyphenols, and offering valuable insights to the study of microorganisms and tea plants.

